# Cognitive-motor exergame training on a labile surface in stroke inpatients: study protocol for a randomized controlled trial

**DOI:** 10.3389/fneur.2024.1402145

**Published:** 2024-06-19

**Authors:** Joel Büttiker, Detlef Marks, Manuel Hanke, Sebastian Ludyga, Petra Marsico, Benjamin Eggimann, Eleftheria Giannouli

**Affiliations:** ^1^Department of Health Sciences and Technology, ETH Zurich, Zurich, Switzerland; ^2^Rehaklinik Zihlschlacht, Centre for Neurological Rehabilitation, Zihlschlacht, Switzerland; ^3^Department of Sport, Exercise and Health, University of Basel, Basel, Switzerland; ^4^Research Department, Swiss Children’s Rehab, University Children’s Hospital Zurich, Zurich, Switzerland; ^5^OST – Eastern Switzerland University of Applied Sciences, Rapperswil, Switzerland

**Keywords:** rehabilitation, neurologic patients, cognition, proprioception, balance, gait, technology-based training, active video games

## Abstract

**Background:**

Cognitive-motor training in form of exergames has been found to be feasible and effective for the improvement of motor and cognitive functioning in older adults and several patient populations. Exergame training under unstable conditions might increase the proprioceptive resources needed and thus might be a superior training approach compared to exergame training on stable ground for stroke patients, who often have proprioceptive deficits.

**Objective:**

Aim of this study is to assess the feasibility and effects of exergame-based cognitive-motor training on a labile platform on physical and cognitive functioning in stroke inpatients.

**Methods:**

This is two-armed pilot randomized controlled trial taking place in an inpatient neurologic rehabilitation clinic. A total of 30 persons that are undergoing inpatient rehabilitation due to a stroke will be randomly assigned to either the intervention group (IG) or the control group (CG). Participants of the IG will receive exergame-based motor-cognitive training on a labile surface, whereas participants of the CG will train on a stable surface. Primary outcome is feasibility comprising measures of adherence, attrition, safety and usability. Secondary outcomes will be measures of cognitive (psychomotor speed, inhibition, selective attention, cognitive flexibility, brain activity) and motor (functional mobility, gait speed, balance, proprioception) functioning.

**Results:**

Data collection started in February 2024 and is expected to be completed by August 2024.

**Conclusion:**

This is the first study looking into exergame training on labile surface in stroke patients. It will give valuable insights into the feasibility and potential added value of this type of training and thus inform further implementation efforts in the context of inpatient rehabilitation.

**Clinical trial registration:**

ClinicalTrials.gov, NCT06296069.

## 1 Introduction

With the growing number of older adults due to the demographic shift, the risk of cardiovascular and neurologic diseases and especially stroke rises (1). Due to an increasing prevalence and a shift to younger age groups, stroke is the second-leading cause of death and third-leading cause of death and disability combined worldwide (2). The inpatient care, rehabilitation and follow up care of stroke patients is over 3% of the value of lost welfare/gross domestic product in certain regions (3). Twenty-six percent of the persons who suffered a stroke remain with limited ability to perform activities of daily living (ADLs) and 50% have reduced mobility due to hemiparesis (4). Post-stroke cognitive impairment (PSCI) is the occurrence of cognitive deterioration after a stroke, which can range from minor impairment to dementia. Studies show that PSCI occurs in up to 60% (cumulative incidence) in the first year (5) as well as 10 years (6) after stroke. PSCI can severely limit the motor and cognitive functioning of the patients and reduce their independence by affecting memory, attention and executive functions (7). Furthermore, the presence of any degree of cognitive impairment (MCI) be a risk factor for falls and other comorbidities of the musculoskeletal system (8).

As a result of the impairment in cognitive and motor functioning after a stroke, the balance ability worsens and gait becomes unsteady. Of all complications following a stroke, falls are one of the most prevalent. Between 14–65% of people with stroke fall at least once during hospitalization and between 37–73% fall during the first six-months after discharge (9). Fall risk is up to two times higher even at later stages after stroke compared to similarly aged individuals (10). Thus, there is also an increased need for interventions in this population.

Balance training is an established form of exercise in people suffering from stroke and other neurological disabilities (11). However, cognitive-motor training is superior to single physical training in improving motor functioning, e.g., gait speed and walking endurance in stroke patients (12). More specifically, compared to sequential (e.g., cycling followed by cognitive training) and simultaneous-additional (e.g., cycling while solving an arithmetical task), simultaneous-incorporated motor-cognitive training (e.g., *any type of training in which the cognitive task is “incorporated” into the motor task,* i.e.*, the cognitive task is a relevant prerequisite to successfully solve the motor-cognitive task*) (13) seems to be the most promising training type for improving gait speed, walking endurance, cadence and stride length in stroke patients (12).

Exergames (video games which are played by body movements) are an excellent tool for the delivery of simultaneous-incorporated cognitive-motor training and they have already been used in the context of several frail and neurologic populations (14–18), including stroke patients (19, 20).

Proprioception is used to stabilize the body by sensing its position in space via the sense of joint and limb positioning. Proprioception training addresses the balance and somatosensory stimulation and can therefore build a possible prevention strategy for further falls and of managing ADLs (21). Combining proprioceptive training with simultaneous cognitive tasks could have additional positive outcomes in stroke rehabilitation. Indeed, a recent systematic review concluded that proprioceptive combined with dual-task exercises stimulate and promote postural balance, gait, and quality of life and reduce the risk of falls in stroke patients compared with traditional rehabilitation programs (22).

There is currently just one study that has looked into the effects of exergame-based cognitive-motor training with the additional proprioceptive stimulation by playing the exergames on a labile platform (23). They found that compared to the training on a stable platform and to a passive control group, training on an instable platform is more effective for the improvement of reactive balance and functional mobility under dual-task conditions in healthy, community-dwelling older adults. The feasibility and effects of this type of exergame training on labile surface and thus rich in proprioceptive stimulation in stroke patients remains unknown.

Therefore, the aim of this study is to assess the feasibility and effects of exergame-based cognitive-motor training on a labile platform on physical and cognitive functioning in stroke inpatients.

We hypothesize that exergame-based cognitive-motor on a labile surface will be feasible within the context of inpatient rehabilitation of stroke patients. In addition, we hypothesize that compared to training on stable surface, training on a labile platform will be more effective for the improvement of motor and cognitive functioning in stroke inpatients.

## 2 Materials and methods

This study protocol was constructed using the SPIRIT reporting guidelines (24).

### 2.1 Study design and procedures

The study is a pilot randomized control trial with two parallel groups; a control group and an intervention group with a 1:1 allocation ratio. The study will only be blinded on pre-measurement. Afterward group assignment blinding is no longer possible because the pre-assessments and training sessions will be carried out by the same study staff. Data collection will be carried out in the neurologic rehabilitation clinic in Zihlschlacht, Switzerland. All study procedures will be performed in accordance with the Declaration of Helsinki. The study protocol was reviewed and approved by the Cantonal Ethics Committee of Eastern Switzerland (EKOS 24/002). Any substantial amendment to the study protocol will have to be approved by the same Ethics Committees and the trial registration at clinicaltrials.gov NCT06296069 will be updated accordingly.

At clinic admission, patients potentially fulfilling the eligibility criteria will be informed in oral and written form about the study and asked if they wish to participate. All interested participants will then be screened for eligibility by the local principal investigator. The included participants will undergo the baseline assessments (T1) and will be subsequently randomly allocated to the intervention or the control group using permuted block randomization with blocks of four. Screening and baseline measurements will be conducted within the first two days upon admission. To minimize physical and cognitive fatigue, a consistent assessment sequence alternating between physical and cognitive tests will be enforced. Participants will be encouraged to ask for a break whenever needed. One day after the T1 measurements, the intervention period will begin. The intervention period will be equal to the length of the stay in the rehabilitation clinic (between 3–4 weeks, according to cantonal/regional regulations and insurance coverage). At the last two days before discharge, post-measurements (T2-measurements) will be performed with all (intervention & control) study participants. Participants will be withdrawn from the study if they develop symptoms or diseases regarded as exclusion criteria during the study.

### 2.2 Participants and eligibility

Primary outcomes of this pilot study are feasibility measures (e.g., adherence, attrition, motivation, enjoyment, adverse events) which do not require an *a priori* sample size calculation. The selected sample size is based on the recommendations of Whitehead et al. (25). Since we are aiming for a future main trial designed with 90% power and two-sided 5% significance and aim to be able to detect medium effect sizes, we will use a sample size of n = 15 per treatment arm. Therefore, 30 participants will be included in the study with 15 participants allocated to each group (intervention or control group). Inclusion criteria are: prescription for inpatient rehabilitation due to a stroke, ability to provide a signed informed consent, age ≥ 50 years, Mini Mental State Examination (MMSE) ≥ 20, ability to stand for at least 3 min without external support. Exclusion criteria are: depending on assistance for ambulation (Functional Ambulation Categories <2), insufficient knowledge of the German language to understand the instructions and games, conservatively treated osteoporotic fractures in the last 16 weeks and presence of any mobility, cognitive, sensory and/or psychiatric limitations or comorbidities which impair the ability to play the exergames and/or conduct the pre-/post assessments.

### 2.3 Interventions

**Figure 1:**
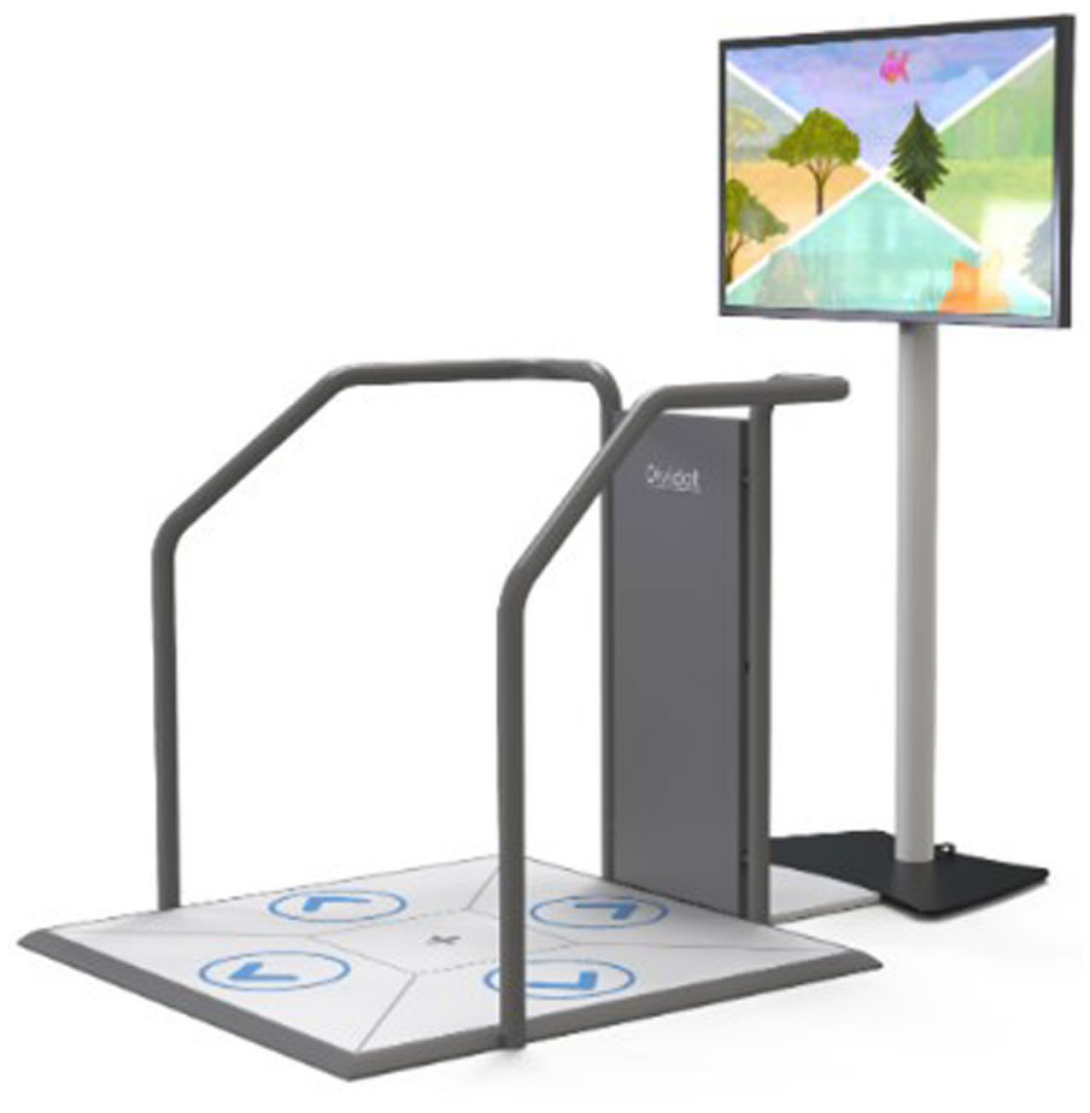
Senso.

**Figure 2:**
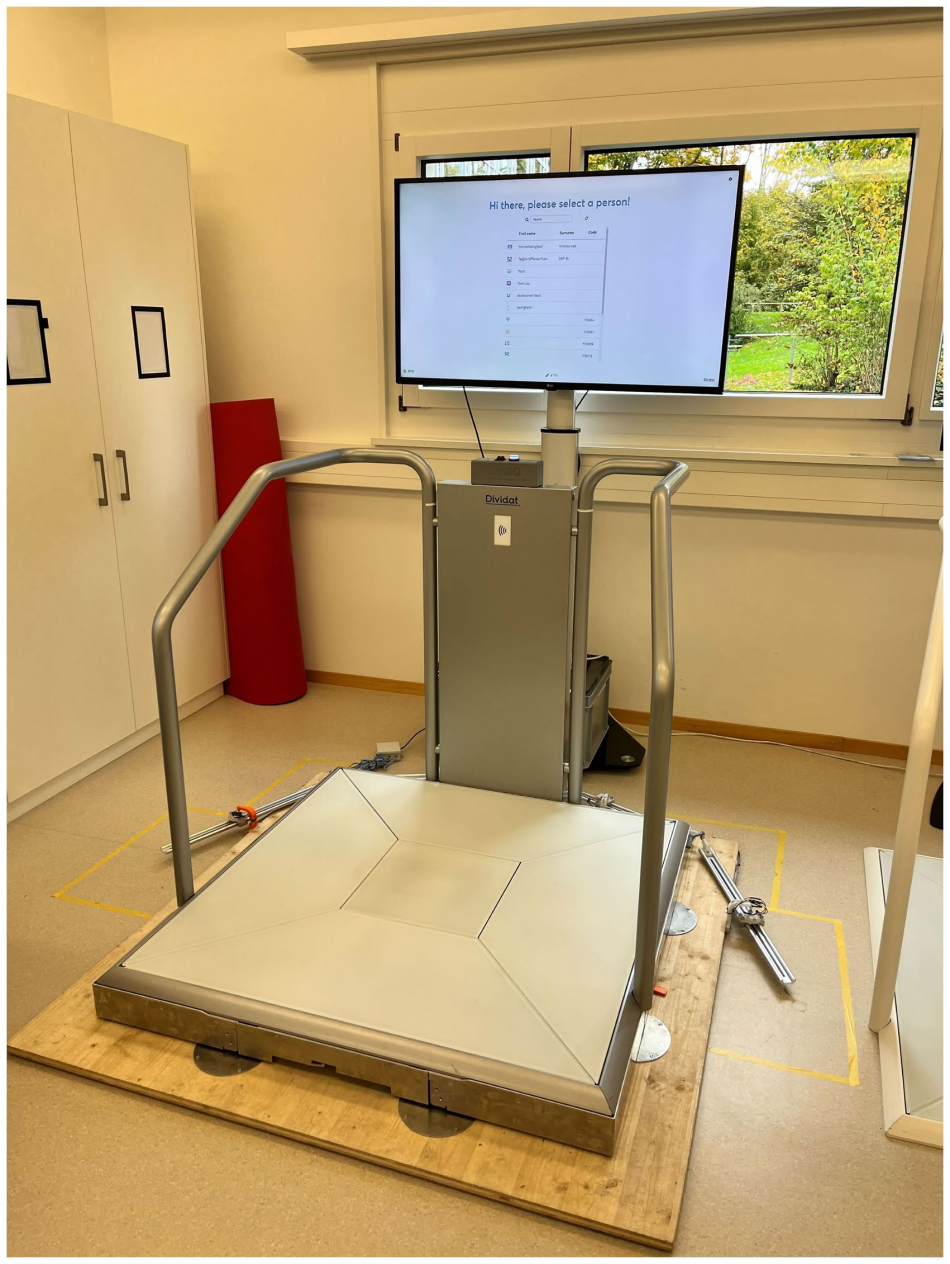
Senso-Swing.

This study will have two arms: a control group for which the conventional treatment during the stay in the inpatient rehabilitation clinic includes a cognitive-motor intervention on a stable surface using the exergame device Senso (Dividat AG, Schindellegi, Switzerland, CE certified, see Figure 1) and an intervention group where the conventional treatment during the stay in the inpatient rehabilitation clinic is extended with an exergame-based cognitive-sensorimotor intervention on an unstable surface by placing the Senso on an unstable surface (Senso-Swing, see Figure 2).

The Senso is a platform for the dynamic recording of steps, weight shifts and other body movements producing forces. The feedback for the user is given visually and auditory by the screen and tactile by vibrating plates. For the labile condition, the Senso is mounted on steel balls, allowing the platform to swing freely along the horizontal plane. There is no movement induced by the platform itself. Sway is only induced when the participant steps and shifts the center of pressure. The degree of instability and movement of the platform can be adjusted by inducing a dampening. The dampening can be set manually, either to on or off. When damping is on, the movement can be reduced by predefined percentages. The maximum displacement of the platform is thereby 100 mm to each side.

The exergames delivered by the Senso specifically target cognitive functions relevant for the successful mastering of activities of daily living, such as executive and attentional functions and physical functions such as balance and coordination. The games are played by conducting body movements, mainly steps in four directions (front, right, left, back) but also body weight shifting. An overview of the 18 existing games and the cognitive/physical domain they train is provided in Supplementary material A.

All training sessions will be supervised by a qualified study/clinic staff who will carefully observe patients while training and aid if necessary.

In general, both groups will conduct the same training program meaning it will have the same volume and the training of participants from both groups will be personalized and designed based on the same progression principles: training duration (15 min training time at the first session up to 28 min in the last session) and game difficulty (starting from rather simple and progressing to more demanding games) (26). In addition to those principles, for the intervention group the degree of instability of the labile platform also will be gradually increased (starting with 75% of the movement damped up until no dampening at the last sessions). The detailed training plan across the 4 weeks of the intervention is presented in Supplementary material B.

After each training session participants will be asked the two questions from the NASA Task Load Index (27) regarding Physical Demand and Mental Demand namely: “How mentally demanding was the training?” and “How physically demanding was the task?.” Answers at the NASA-TLX are in a scale of 1 to 20. Any answer between 10–15 is considered the “sweet spot” (20) for which the pre-defined training plan will be applied as described in Supplementary material C.

Since training intensity is decisive for the success of the intervention, in case participants rate either of the questions between 1–9 or 16–20 and in order to decrease the risk of losing the potential benefit or, respectively, the safety risk (28) the next training session will be adapted either by increasing/decreasing the duration of the next training session or by increasing/decreasing difficulty of the exergames.

**Figure 3:**
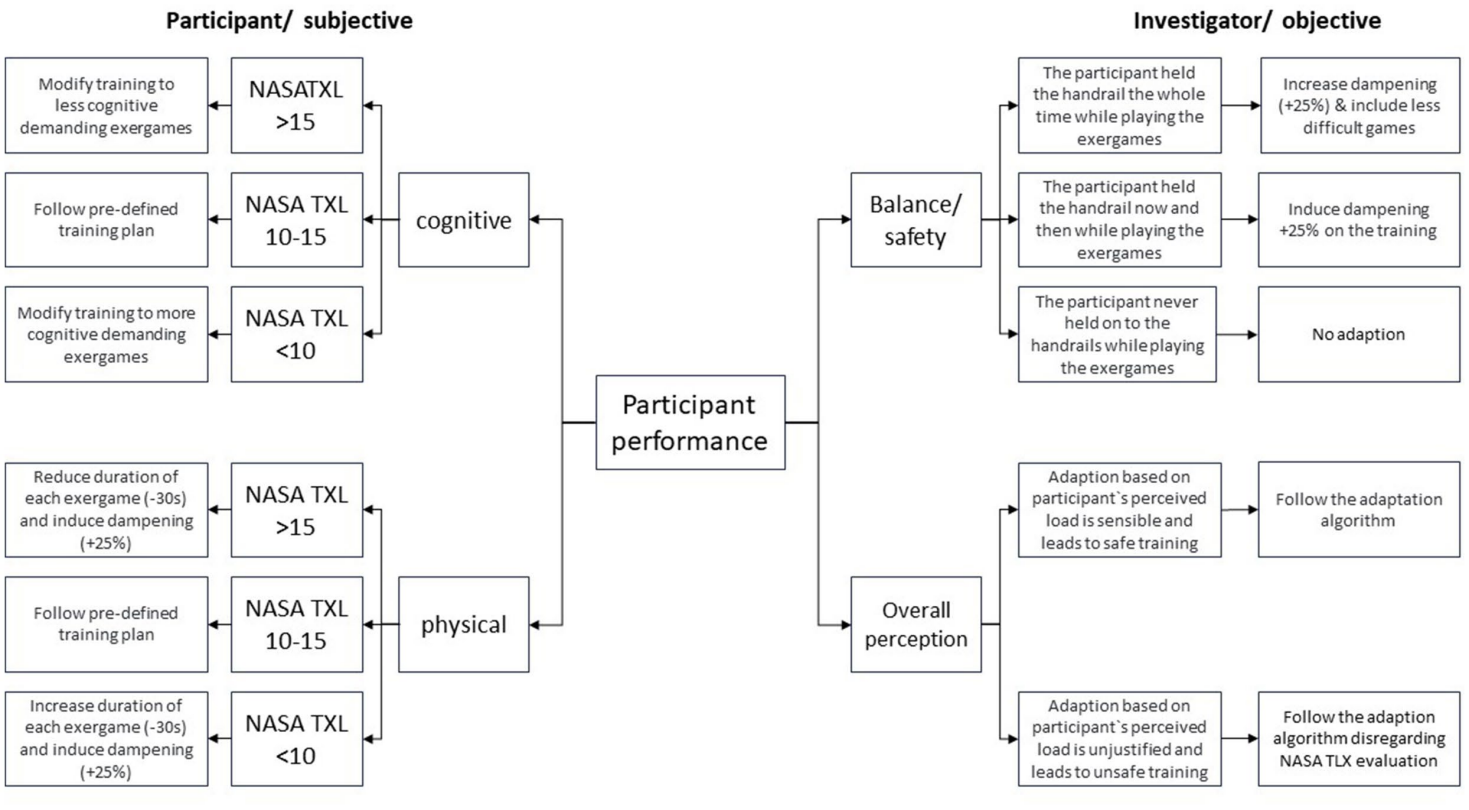
Training adaptation concept.

Adaptations of the pre-defined plan can also be made based on the trainer’s evaluation of participant’s safety and training success, meaning they can decide at their own discretion whether the adaptation (based on participant’s perceptions) is justified or whether patients are at risk due to overestimating themselves. Figure 3 provides a detailed description of the training progression adaptation guidelines.

### 2.4 Outcomes

#### 2.4.1 Primary outcomes

Primary outcome of this study is feasibility, defined as an umbrella term comprising several measures of acceptance and safety. They are hereinafter described.

##### 2.4.1.1 Safety

Adverse events throughout the intervention period will be protocolled and categorized into serious and non-serious as well as intervention-related and intervention-unrelated. All serious intervention-related adverse events will be reported to the ethics committee.

##### 2.4.1.2 Attrition

The number of participants that dropped-out during the trial will be recorded for both groups. Drop-out reasons will be documented if available. Considering the median rate for attrition in preventive interventions for older adults in community settings for clinical trials (29) a 10% attrition rate can be deemed acceptable.

##### 2.4.1.3 Adherence

Attendance of each training session will be recorded in an attendance protocol by the study investigator. Average adherence rates across the intervention period will be calculated. The adherence will be calculated as the mean adherence rate (%) = number of training sessions attended / total number of training sessions offered. A review by Nyman and Victor (29) reveals a 50% attendance rate to preventive interventions for older adults in clinical trials. Nevertheless, in this study, an 80% adherence rate for the training sessions is set as the definition for being adherent to the training program.

##### 2.4.1.4 Usability

The overall usability of the exergame training system will be assessed post-intervention with the System Usability Scale (SUS) (30). The SUS provides a global view of subjective assessments of usability such as the need for support, training and complexity. We use a German translation which has already been used in large studies (31). The scale was successfully applied in previous studies looking into the feasibility of exergame training in the context of inpatient rehabilitation of older adults and neurologic populations (15, 16). Based on the verbal categorization/adjective rating of Bangor (32) we expect a SUS score of at least 70 to have an “acceptable” solution (52 = ok, 73 = good, 85 = excellent, 100 = best imaginable).

##### 2.4.1.5 Training load

Physical and cognitive load of each training session will be assessed using the Nasa Task Load Index (NASA-TLX). The NASA-TLX is a self-report, multidimensional assessment tool that rates perceived workload in order to assess a task, a system, or other aspects of performance (in this case the exergame training). Originally it consists of five subscales, but this study uses only: Mental Demand and Physical Demand. Answers will be in a scale of 1 to 20 (33). Answers will be presented descriptively for each training session as well as aggregated by averaging values across all training sessions (though separately for the physical and cognitive workload).

##### 2.4.1.6 Enjoyment

To assess training enjoyment, we will use the Exergame Enjoyment Questionnaire (EEQ). This questionnaire consists of a 5-point Likert scale which you reflect your enjoyment and feelings from “do not agree at all” to “I fully agree” while training on the device. The score will be calculated by adding up the points of each question resulting in a minimum of 20 points and a maximum score of 100. The higher the score the greater the exergame enjoyment (34).

##### 2.4.1.7 User experience

Several questions specifically tailored to this study regarding perceived safety, perceived positive effects, intention to recommend etc. will be used. Most questions will have a 7step Likert Scale answers. However, there will also be two open ended questions asking for any positive/negative feedback and other general remarks by the participants.

##### 2.4.1.8 Training goals

Personal goals regarding rehabilitation/training will be assessed with the Goal Attainment Scale *(GAS)*. The GAS is an individual approach to defining and evaluating personal rehabilitation goals (35, 36). The goals will be defined at the beginning of the intervention and will be reevaluated in the middle (after 8 trainings) and at the end of the intervention period. The scale consists of a five-point rating of the achievement of the specified goals. A score of 0 corresponds to the expected improvement or achievement of the predefined goal. A negative score of −1 or −2 is considered worse than expected. A positive score of 1 and 2 is given when the goal is achieved even better than expected. Interpersonal scores for the three time-points will be evaluated descriptively for each participant separately.

The feasibility of the intervention is pre-determined using the following feasibility criteria:

- no intervention-related adverse events.
- maximum 10% attrition rate.
- at least 70% adherence rate.

If all three criteria are met, we deem the intervention to be feasible. If one to two criteria are not met, the intervention is feasible but needs modifications. If none of the criteria are met, the intervention will be deemed not feasible.

#### 2.4.2 Secondary outcomes

Secondary outcomes of this study are measures of physical and cognitive functioning.

##### 2.4.2.1 Cognitive functions

###### 2.4.2.1.1 Cognitive flexibility

Cognitive flexibility will be assessed using the Trail making test (TMT): The TMT is a widely used neuropsychological test only requiring paper and pencil (37–39) and has two parts, TMT.A and TMT.B. Circled numbers from 1 to 25 are allocated randomly on a sheet which participants have to connect in the right order (TMT.A). At TMT.B, circled numbers and letters are randomly allocated on a sheet and the participants have to connect circled numbers and letters in the right order and in alternating manner. The required time to complete each task as well as the difference between the scores TMT.B-TMT.A measured in both parts will be evaluated.

###### 2.4.2.1.2 Psychomotor speed

**Figure 4:**
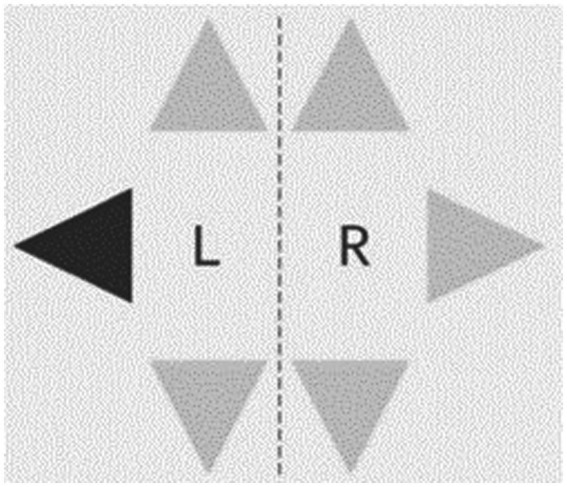
Display of step reaction time test.

The *Step Reaction Time Test* will be conducted using the Dividat Senso and it measures psychomotor speed in terms of reaction to visual stimuli using the lower extremities in 6 directions (front right, front left, right, left, back right & back left). There are six light grey triangles on the screen and each time one of then turns black, participants need to step as quickly as possible in the respective direction (Figure 4). Average reaction time across all stimuli will be used for analyses.

###### 2.4.2.1.3 Selective attention

**Figure 5:**
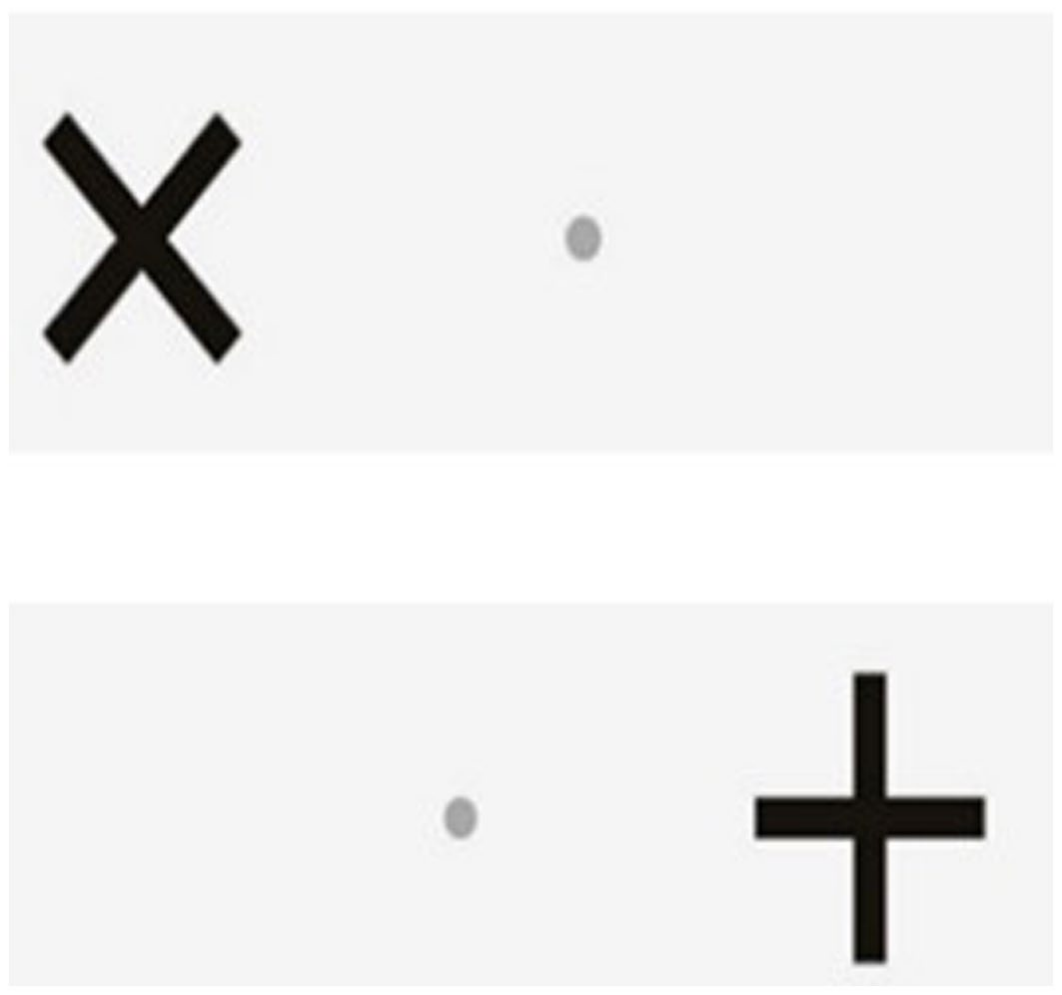
Display of the Go/No-Go test.

The Go/No-Go test will be conducted using the Dividat Senso and it measures selective attention and inhibition (40). Participants fixate on a small grey dot in the middle of the screen. Crosses (+) and Xs (X) appear on the right and left side of the grey dot in a randomised order. The task is to ignore the + and just conduct a step as quickly as possible in the direction that an (X) appears (Figure 5).

###### 2.4.2.1.4 Inhibition and brain activity

The Stroop Test assesses inhibition (cognitive interference). The interference occurs when a specific stimulus is impeded by a second stimulus attribute, known as Stroop Effect (41). A computer-based version of the Stroop Colour-Word will be conducted to assess the inhibitory component of executive functioning (42–44). In compatible trials of the task, colour words are presented in the same colour (e.g., “green” printed in green), whereas in incompatible trials, colour words are presented appearing in a different colour (e.g., “yellow” printed in blue). Participants are instructed to press a button corresponding to the word meaning, ignoring the colour of ink the word is written in. Before the test, 4 examples are shown and a practice round is administered, which includes 12 trials with feedback on the response. Afterwards, compatible and incompatible trials are presented in 8 alternating blocks (with 12 trials each), which are interspersed by a recovery period. On each block, colour words are presented for 350 milliseconds (ms) on black background and responses are allowed within a 1,250 ms time window. To avoid habituation, the inter-stimulus interval varies randomly between 900 and 1,100 ms. Reaction time in ms on response-correct trials and accuracy in % is extracted separately for compatible and incompatible trials. Interference in ms is calculated as the difference in reaction time (on trials with correct responses) between compatible and incompatible trials. The Stroop Test will be coupled with a functional near-infrared spectroscopy (fNIRS) system (NIRx Medical Technologies, NIRSport2, Berlin, Germany) to assess changes in cortical haemodynamics during the cognitive test. The systems combines 8 light sources and 7 detectors, which are evenly distributed over the prefrontal cortex, resulting in 20 measurement channels. Based on neurovascular coupling, these measured changes allow conclusions on neural activity in this brain area of interest (45). Outcomes are the peak and average changes from resting baseline concentration of oxygenated (HbO2) and deoxygenated haemoglobin (HHb) during compatible and incompatible test blocks, respectively.

##### 2.4.2.2 Physical functions

###### 2.4.2.2.1 Functional mobility

Functional mobility will be measured using the instrumented version of the Timed Up and *Go Test (iTUG)*. The iTUG (46), is based on the “normal” TUG test developed by Podsiadlo and colleagues 1991 (47). It is an easy-to-do test that requires only a chair and a stopwatch. Four inertial sensor units (Opal, APDM, Oregon, United States) are attached to the participant’s body with elastic straps. At the start signal participants must stand up from a chair, walk 3 m at a comfortable walking speed, come back and sit down on the chair again. Time to complete the task as well as several other performance metrics from all the test’s phases (sit-to-stand transition, gait, turn and turn-to-sit transitions) are computed with the Software “Mobility Lab 2^®^; Oregon, Version 2.0.0.201903301644,” that comes along with the inertial sensor system. A dual-task condition will also be conducted. In the dual-task condition, a second (cognitive) task is added; participants have to count backwards in steps of three from a random given number between 200 and 250 while they are performing the test (“serial threes”). Following outcome measures will be used for further analyses for the single task and the dual-task conditions respectively: total duration, sit-to-stand duration, turn velocity, turn-to-sit duration. Additionally, relative dual task costs (DTC) of walking as percentage of loss relative to the single-task walking performance, according to the formula DTC [%] = 100 * (single-task score − dual-task score)/single-task score (48) will be calculated.

###### 2.4.2.2.2 Coordination

Motor coordination is assessed using the 4 Step Square test (4SST). The 4SST assess a person’s ability to step as quickly as possible in all 4 directions: forward, backward and sidewards. At the start, the participant stands in Square 1, facing Square 2 and will step clockwise over every Square until Square 4 and anti-clockwise back to Square 1. Time is measured to complete this task (49).

###### 2.4.2.2.3 Dynamic balance

**Figure 6:**
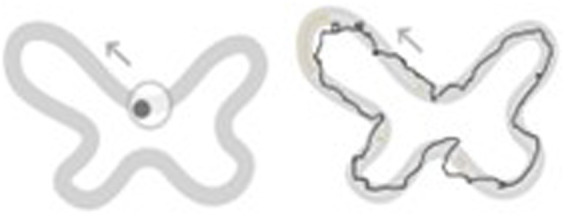
Display of shape tracking test.

Dynamic balance is assessed with the Shape Tracking Test. Participants are asked to move their center of pressure (COP) displacement by bending or rotating their body without moving the feet, so that they remain within the track that is shown on the screen (see Figure 6).

###### 2.4.2.2.4 Static balance

*Postural Sway* will be assessed with the iSway test (50) of the APDM inertial sensor system. One inertial sensor unit (Opal, APDM, Oregon USA) is attached to the participant’s body (lower back) with an elastic strap. Participants are required to stand as still as possible for 30 s. Several center of pressure (COP) displacement measures are computed with the Software “Mobility Lab 2^®^; Oregon, Version 2.0.0.201903301644,” that comes along with the inertial sensor system. Mean displacement velocity and sway area will be used for further analyses.

###### 2.4.2.2.5 Gait

Gait Analysis will be conducted with the iWalk test of the APDM inertial sensor system. Four inertial sensor units (Opal, APDM, Oregon United States) (two at the feet, one at the lower back and one on the chest) are attached to the participant’s body with elastic straps. Participants are required to walk for 2 min as fast as possible (but without running). Several gait performances metrics are computed with the software “Mobility Lab 2^®^; Oregon, Version 2.0.0.201903301644” (51), that comes along with the inertial sensor system. Stride length, stride velocity, and gait variability will be used for further analyses.

###### 2.4.2.2.6 Leg proprioception

Leg proprioception will be assessed using the Dynamic Position Test of the ProMeTo-System. In this test, the examiner will move participants joints (extension or flexion) into several positions/angles. Participants will be asked to memorize and replicate exactly the position that the examiner specified without visual control. Three different joints are tested: hip, knee and ankle. The range of motion (ROM) of every joint is measured by moving the joint two times in either full internal rotation and external rotation for the hip or extension and flexion in the knee prior to the onset of the test. In the ankle joint the plantarflexion and dorsiflexion ROM will be measured. Inertial sensors (Shimmer Research Ltd., Dublin, Ireland) provide the angle difference (in degrees) between the position given by the test leader and the position imitated as accurately as possible by the participant. Average angle differences for each joint will be used for further analyses.

Because there are currently no reference values and/or standard error measurement values for this population, this test will be repeated twice before the onset of the intervention (once during the pre-assessment, together with all the rest of the pre-port assessments and once again one day later, just before starting the first training session) in order to calculate its test–retest reliability and assessment error (minimum detectable difference) for this population.

###### 2.4.2.2.7 Balance confidence

The German version of the Activity-specific balance confidence scale (ABC-D) will be used to assess balance confidence in various activities in older people (52). The questionnaire uses an answer scale from 0 to 100% about the confidence of maintaining balance by activities. An answer of 0% indicates no confidence in conducting the activity and 100% suggests full confidence in performing the activity. In total 16 questions will be asked, and the total mean scores will be calculated.

###### 2.4.2.2.8 Gait confidence

The German version of the Modified gait efficacy scale (mGES-D) will be used to assess perception of confidence in walking under challenging circumstances. It is a 10-item questionnaire on a 10-point Likert scale. 1 means no confidence; 10 means full confidence. 100 points means complete confidence in every task (53).

#### 2.4.3 Demographic data

The following demographic data will be collected to further describe the study population: age, years of education, body weight, body height, NIH Stroke Severity Scale (54), main symptoms, time since stroke, comorbidities using the Cumulative Illness Rating Scale (CIRS) (55) and further treatments received during the intervention period.

#### 2.4.4 Statistical analyses

All statistical procedures will be conducted with the IBM SPSS statistics software or R (RStudio, Boston, MA, United States). For demographics as well as training adherence and compliance, all collected data will be included (i.e., including data of dropouts up to the time point of their withdrawal). For all further analyses, only data of participants with an adherence ≥70% will be analyzed (per protocol analysis). A separate descriptive analysis of data from withdrawn participants who terminated the intervention prematurely or had to be excluded during the study or participants with adherence <70% will also be provided. Data will be reported as mean (SD) values for continuous parametric data and median (IQR) values for continuous nonparametric data. Data will be tested for normal distribution using Shapiro-Wilks Test and Q-Q-plots as well as for homogeneity of variance using Levene test. General level of significance used is established as *p* = 0.05. For the physical and cognitive tests that serve as secondary outcomes and are assessed in a pre-post manner, a two-way repeated measures ANOVA with group assignment (control vs. intervention group) as between subject factor and time-point (pre- vs. post-training) as within factor will be conducted. In case length of stay (and thus number of training sessions) vary across participants, we will conduct repeat our analyses controlling for this parameter. In order to determine the effects of the outcomes, effect sizes will be calculated for all primary and secondary outcomes. If any of the assumptions for parametric testing is not met, the non-parametric alternative (Friedman’s ANOVA) will be used. If the two-way mixed ANOVA or Friedman’s ANOVA report a significant group, time or interaction effect, data will be further analyzed using post-hoc tests. To calculate effect sizes of intragroup differences between post- and baseline measurements, a dependent T-test or its non-parametric equivalent (Wilcoxon signed rank test) will be used. The effect size will be interpreted using benchmarks describing the effect size as small (r ≥ 0.01), medium (r ≥ 0.3), or large (r ≥ 0.5) (56).

## 3 Results

Data collection is expected to start in February 2024 and to be completed in August 2024.

## 4 Discussion

The goal of this pilot RCT is to evaluate the feasibility and effects of an exergame-based cognitive-motor training intervention on a labile surface in stroke inpatients. We expect the intervention to be feasible and more effective in improving motor and cognitive functioning compared to training on a stable surface.

Previous studies using the same exergame device in the same setting (inpatient rehabilitation) with geriatric and Parkinson’s inpatients reported no adverse events, very high adherence and enjoyment levels as well as significant time-group interaction effects for, e.g., gait speed, balance, psychomotor speed and inhibition (15, 16). However, exergame training on a labile surface has not been investigated neither for this population (stroke patients) nor in this setting (inpatient rehabilitation). Therefore, we extend previous intervention approaches by increasing proprioceptive stimulation during training and consequently training effects.

The main challenge we anticipate is low recruitment rate. Inpatient clinics (at least in Switzerland) offer a wide variety of therapies which are offered in a very intensive manner; patients can have up to 1.5 h of therapy per day. This can lead to participation in the study be seen as a burden. Moreover, the study population (persons in the early-subacute phase) is extremely heterogeneous which can affect interpretation of the effect results. What is more, the study will take place in an inpatient rehabilitation clinic and thus training volume will not correspond to the current training recommendations for stroke patients. After a systematic review, healthy elderly people should perform exergame training two to three times per week for 45–60 min for 12 weeks and more to improve cognition (57). In addition, this study’s results will not be generalizable to other stages of the disease (e.g., chronic stroke) or settings (e.g., outpatient rehabilitation). However, examining the feasibility of this intervention within the scope of this study will give valuable information about whether or not such training can be integrated in the inpatient rehabilitation therapy plans. In addition, if found effective, this study would be the foundation for a larger study that is powered to detect effects. Most importantly, the information and knowledge gained from this project will help to adapt and further develop digital solutions and technologies to support patients, therapists, geriatric rehabilitation and potentially the whole health care system.

## Author contributions

JB: Project administration, Writing – original draft, Writing – review & editing. DM: Investigation, Methodology, Project administration, Resources, Writing – review & editing. MH: Resources, Software, Writing – review & editing. SL: Resources, Software, Writing – review & editing. PM: Resources, Software, Writing – review & editing. BE: Resources, Software, Writing – review & editing. EG: Conceptualization, Funding acquisition, Methodology, Project administration, Resources, Supervision, Writing – review & editing.
