# Supplementary material for: Cognitive-motor exergame training on a labile surface in stroke inpatients: study protocol for a randomized controlled trial
Source: Front Neurol. 2024 Jun 19;15:1402145. doi: 10.3389/fneur.2024.1402145 (PMC11223001; doi:10.3389/fneur.2024.1402145)
Supplement: Supplementary file 1 [file Table_1.pdf]

Supplementary Material

Cognitive-motor exergame training on a labile surface in stroke inpatients:

study protocol for a randomized controlled trial

1 Supplementary Material A: Exergames

Game Name	Main Trained Functions	Training Goals	Task/Instruction
**Simple** 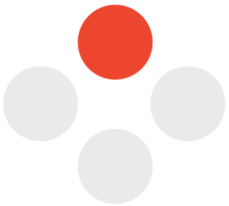	Psychomotor speed	The training game “Simple” trains the attentional focus - the ability to concentrate and focus on stimuli. It also trains the psychomotor processing speed and responsiveness, i.e. to process and react to sensory stimuli as quickly as possible.	*Below are four circles. One of them will turn red. As soon as a circle turns red, take a step in that direction as quickly as possible.*
**Birds** 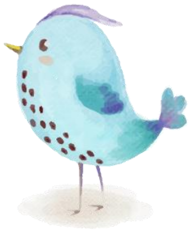	Selective attention	The training game "Birds" trains selective attention - the ability to react to certain (relevant) stimuli and to be able to ignore others (irrelevant).	*Return the feather in the middle to the correct bird. To do this, take a step to the bird that matches the displayed feather. Take the steps as quickly as possible.*
**Ski** 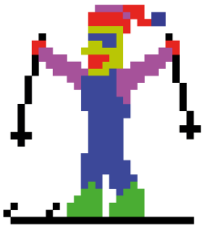	Balance control and weight shifting	The training game "Ski" trains balance control as well as weight shifting.	*Control the skier by shifting your weight. Avoid trees, stones and snow monsters!*
**Hexagon** 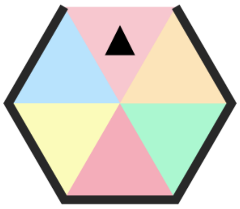	Visuospatial orientation and mental rotation	The training game "Hexagon" trains spatial cognition, visuo-spatial orientation and mental rotation.	*Dodge the walls of the hexagon with steps to the left and right.*

**Targets** 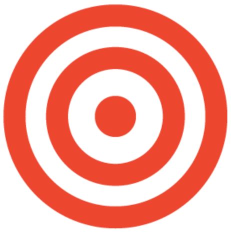	Divided attention, planning, foot placement accuracy and timing	The training game "Targets" trains forward thinking and targeted reaction with correct timing.	*Hit the flying balls in the middle of the targets with one step in the respective direction.*
**Divided** 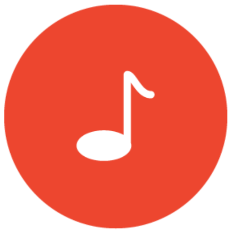	Divided attention	The training game "Divided" trains the divided attention - the ability to process and react to several stimuli simultaneously, especially when the stimuli are presented in different modalities (visual, auditory, tactile).	*Below are four circles. One of them will turn red. As soon as a circle turns red, take a step in that direction as quickly as possible. You will also hear sounds. With a high tone you take a step forward, with a low tone you take a step backwards.*
**Simon** 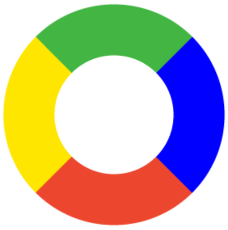	Short-term memory/memory span	The training game "Simon" trains the short-term memory - the ability to remember information (e.g., sequences) at short notice.	*In the following, a tone is assigned to each answer key/step direction. You will be shown a sequence of steps/tones, which you must copy in the correct order. The sequence of steps/tones begins with one element and will then become longer and longer. If you make a mistake, a new sequence begins (start with one element).*
**Flexi**	Cognitive flexibility	The training game "Flexi" trains	*A: There is a number in the*

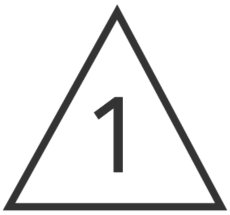		cognitive flexibility - the ability to alternate attention allocation between different stimuli and concepts.	*middle of the screen. In addition, other numbers appear around. Your task is, starting from the number in the middle, to take a step in the direction of the next higher number.*<br><br>*B: In addition, a figure appears around the number. A step must be taken in the direction of the next higher number with the opposite pattern.*
**Lumina**<br>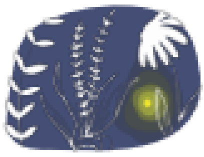	Static balance, relaxation	The Lumina training game trains the static balance with the main focus on recovery and relaxation. The gentle flying through different worlds is accompanied by relaxing music. Through the targeted weight shifting in all four directions, the fine control of balance is balance is promoted.	*Stand hip-width apart on the exercise area. With slight shifting your body weight you steer the firefly to the right or left. You can change the flight altitude upwards if you shift your weight on your heels. The firefly moves downwards when you stand on your tips of your feet. Dots of light can be collected, which appear on the flight path.*
**Habitats**	Selective attention and inhibition	The training game "Habitats" trains selective attention - the ability to react to certain stimuli and to be able to ignore other	*Animals move across the four landscapes in the picture. If an animal does not appear in its*

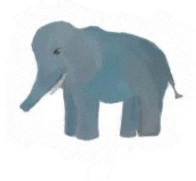		stimuli. Provoked reactions must be suppressed.	*usual environment, take a step in this direction. Do not try to disturb animals in their natural habitat.*
**Rocket** 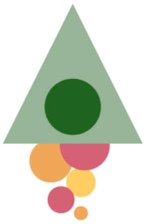	Cardiovascular fitness	The training game "Rocket" trains the cardiovascular system through short, high-intensity stepping bouts.	*Take steps on the training plate to fly the rocket through the universe. A green arrow in front of the rocket indicates that you should increase your stepping speed. A red bar behind the rocket indicates that you are too fast and should slow down your stepping speed.*
**Ladybug** 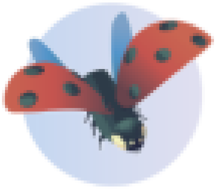	Weight shifting and action planning	The training game «Ladybug» trains forward thinking action planning and static balance.	*Stand in the center with your feet more than hip-width apart. Control the ladybug by leaning only to the right and left. Your goal is to collect the flowers and avoid obstacles. Some flowers glisten in the dew. Focus on them to get a bonus.*

**Cloudy**<br>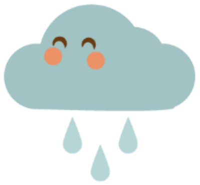	Static balance	The training game "Cloudy" trains static balance and fine adjustment of movement control. It requires the targeted and controlled shifting of the body's center of gravity. Moreover, basic attentional functions are trained.	*Water the thirsty flowers. Move the rain cloud over the flowerpots by taking steps to the right or left side. Be careful with the steps, a light tap is enough.*<br><br>*Tip: Try to water the bonus flower, which is shown below.*
**Sam's Garden**<br>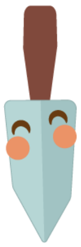	Dynamic balance	The training game "Sam's Garden" trains various aspects of coordination and balance as well as cognition.<br><br>**Motor functions:** dynamic balance and coordination of the lower extremities (based on the processes of daily walking), goal-oriented movement in space<br><br>**Cognitive functions:** In addition to basic attention, the game requires simultaneous processing of a relatively complex virtual environment with multiple target stimuli, higher brain functions are required.	*Harvest the vegetables in the garden and weed using the shovel. By walking around on the plate, you move the shovel to the place where the vegetables or weeds are.*<br><br>***Vegetables: Remain** on the vegetables until they are harvested.*<br><br>***Weeds: Jump** on the weeds until they disappear.*<br><br>*Tip: Try to harvest the bonus vegetables, which are shown below.*
**Evolve**<br>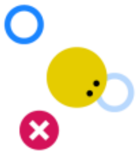	Static balance, selective attention	The training game Evolve trains the control and various aspects of cognition such as selective attention - the ability to react to certain stimuli and ignore	*Blue rings, red dots, and a yellow figure are displayed on the screen. By shifting the center of pressure, the yellow figure can*

		other stimuli, as well as thinking ahead and action planning.	*be controlled to catch the blue rings while avoiding the red dots.*
**Drops**<br>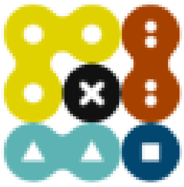	Action planning,<br><br>orientation	The training game "Drops" trains<br><br>thinking, visual-spatial orientation and mental rotation skills.	*Colored drops fall as a pair from above. When drops land near a drop of the same color, they join together to form a group. Gather groups of four or more drops to create space and score points. Step left or right to move the pair sideways. Step forward to rotate the pair. Step backward*<br><br>*to make it fall faster. You get more points if several groups dissolve when you place a single pair of drops.*
**Snake**<br>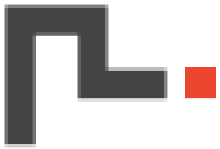	Action planning,<br><br>orientation	The training game "Snake" trains the<br><br>planning and orientation ability in a room (2D) (visual-spatial orientation).	*A snake is displayed on the*<br><br>*screen which has to be*<br><br>*controlled by stepping on the*<br><br>*four plates. The aim is to "feed" the snake with as many red squares as possible which are also displayed on the*

			screen. The snake can also move beyond the edge of the screen.
**Flaneur** 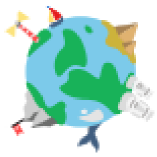	Endurance	The “Flaneur” training game is a simple<br><br>endurance training.	*This game is a virtual walking tour with a street view. Steps on the middle plate have to be made to go for a walk through Zurich. The faster the steps are made, the faster the virtual walking speed.*

2 Supplementary Material B: Training plan

Session / Games	**Rocket**	**Ladybug**	**Birds**	**Divided**	**Simon**	**Hexagon**	**Habitats**	**Targets**	**Flexi**	**Ski**	**Sam's Garden**	**Evolve**	**Cloudy**	**Drops**	**Snake**	**Simple**	**Lumina**	**Flaneur**	**Net gaming time**
1	1min (100%)	2x1min (100%)	2min (100%)								2x1min (100%)		2min (100%)		2min (100%)	2x1min (100%)		2min (100%)	15min
2	1min (100%)	2x1min (100%)	2min (100%)								2x1min (100%)		2min (100%)		2min (100%)	2x1min (100%)		2min (100%)	15min
3	1min (75%)	2x1min 75%)	2min (75%)					2x1min (75%)			2x1min (75%)		2min (75%)		2min (75%)	2x1min (75%)		2min (75%)	17min
4	1min (75%)	2x1min (75%)	2min (75%)					2x1min (75%)			2x1min (75%)		2min (75%)		2min (75%)	2x1min (75%)		2min (75%)	17min
5	1min (50%)	2x1min (50%)	2min (50%)					2x1min (50%)			2x1min (50%)		3min (50%)		2min (50%)	2x1min (50%)		2min (50%)	18min
6	1min (50%)	2x1min (50%)	2min (50%)					2x1min (50%)			2x1min (50%)		3min (50%)		2min (50%)	2x1min (50%)		2min (50%)	18min
7	1min (25%)	2min (25%)	2min (25%)					2min (25%)		2min (25%)	2min (25%)	2min (25%)	3min (25%)		2min (50%)	2min (25%)		2min (25%)	22min
8	1min	2min	2min					2min		2min	2min	2min	3min		2min	2min		2min	22min

	(25%)	(25%)	(25%)					(25%)		(25%)	(25%)	(25%)	(25%)		(25%)	(25%)		(25%)	
9	1min<br>(0%)	3min<br>(0%)		2min<br>(0%)	2min<br>(0%)		2min<br>(0%)	2min<br>(0%)		2min<br>(0%)		2min<br>(0%)		1min<br>(0%)		2min<br>(0%)	1min<br>(0%)	2min<br>(0%)	22min
10	1min<br>(0%)	3min<br>(0%)		2min<br>(0%)	2min<br>(0%)		2min<br>(0%)	2min<br>(0%)		2min<br>(0%)		2min<br>(0%)		1min<br>(0%)		2min<br>(0%)	1min<br>(0%)	2min<br>(0%)	22min
11	1min<br>(0%)			2min<br>(0%)	2min<br>(0%)	1min<br>(0%)	3min<br>(0%)	3min<br>(0%)	2min<br>(0%)	2min<br>(0%)		3min<br>(0%)		2min<br>(0%)			1min<br>(0%)	2min<br>(0%)	24min
12	1min<br>(0%)			2min<br>(0%)	2min<br>(25%)	1min<br>(0%)	3min<br>(0%)	3min<br>(0%)	2min<br>(0%)	2min<br>(0%)		3min<br>(0%)		2min<br>(0%)			1min<br>(0%)	2min<br>(0%)	24min
13	1min<br>(0%)			2min<br>(0%)	2min<br>(0%)	1min<br>(0%)	3min<br>(0%)	3min<br>(0%)	3min<br>(0%)	2min<br>(0%)		3min<br>(0%)		2min<br>(0%)			1min<br>(0%)	2min<br>(0%)	25min
14	1min<br>(0%)			2min<br>(0%)	2min<br>(0%)	1min<br>(0%)	3min<br>(0%)	3min<br>(0%)	3min<br>(25%)	2min<br>(0%)		3min<br>(0%)		2min<br>(0%)			1min<br>(0%)	2min<br>(0%)	25min
15	1min<br>(0%)			3min<br>(0%)	2min<br>(0%)	2min<br>(0%)	3min<br>(0%)	3min<br>(0%)	3min<br>(0%)		2min<br>(0%)	3min<br>(0%)		2min<br>(0%)			1min<br>(0%)	2min<br>(0%)	28min
16	1min<br>(0%)			3min<br>(0%)	2min<br>(0%)	2min<br>(0%)	3min<br>(0%)	3min<br>(0%)	3min<br>(0%)		2min<br>(0%)	3min<br>(0%)		2min<br>(0%)			1min<br>(0%)	2min<br>(0%)	28min
17	1min				3min	3min	3min	3min	3min		3min	3min		2min			2min	2min	28min

	(0%)				(0%)	(0%)	(0%)	(0%)	(0%)		(0%)	(0%)		(0%)			(0%)	(0%)	
18	1min (0%)				3min (0%)	3min (0%)	3min (0%)	3min (0%)	3min (0%)		3min (0%)	3min (0%)		2min (0%)			2min (0%)	2min (0%)	28min
19	1min (0%)				3min (0%)	3min (0%)	3min (0%)	3min (0%)	3min (0%)		3min (0%)	3min (0%)		2min (0%)			2min (0%)	2min (0%)	28min
20	1min (0%)				3min (0%)	3min (0%)	3min (0%)	3min (0%)	4min (0%)		3min (0%)	3min (0%)		3min (0%)			3min (0%)	2min (0%)	28min

% is induced dampening effect of movement of the platform: 100%=No movement possible/100%; 0%=No dampening at all, full movement possible

3 Supplementary material C: Exergame

Exergame types and difficulty level categorization

Most of the available exergames are meant to train motor and cognitive functioning simultaneously, i.e. there are several cognitive tasks that require several motor responses. For the purpose of defining training progression, their categorization was done based on the motor and/or cognitive function that is primarily addressed. Thus, the exergames that divided into three categories:

1. Step reaction games: Simple, Target, Snake, Hexagon, Drops, Birds, Habitats, Divided, Flexi)
2. Weight shifting/balance games: Cloudy, Lumina, Ski, Ladybug, Ski, Evolve, Sam`s Garden
3. Memory games (for which reaction time is irrelevant): Simon
4. Endurance games: Rockets

The difficulty level inside those 3 categories is defined according to level of movement needed and challenge of the cognitive task.
